# Diagnostic value of computed tomography plus magnetic resonance imaging in assessing the benign and malignant nature of vertebral compression fractures

**DOI:** 10.3389/fmed.2026.1759764

**Published:** 2026-03-09

**Authors:** Peng Zhao, Xianghong Meng, Man Sun, Yuqiao Zhong, Shan Zhu

**Affiliations:** Radiology Department 2, Tianjin Hospital, Tianjin, China

**Keywords:** apparent diffusion coefficient, computed tomography, diagnosis, magnetic resonance imaging, vertebral compression fractures

## Abstract

**Objective:**

To explore the diagnostic efficacy of combining computed tomography (CT) with magnetic resonance imaging (MRI) in determining whether vertebral compression fractures (VCFs) are benign or malignant.

**Methods:**

From January 2020 to January 2025, 150 patients with single vertebral compression fractures who were admitted to our hospital were selected as study participants. According to the pathological findings, they were divided into the benign group (76 cases) and the malignant group (74 cases). All patients underwent MRI and CT examinations. The efficacy of the combined use of these two methods and the combined detection in differentiating the nature of vertebral compression fractures was analyzed.

**Results:**

Significant differences were observed between the two groups in MRI signs such as the degree of vertebral body compression, the range of vertebral body lesions, the location of the lesions, the post-compression vertebral body morphology, pedicle morphology, the vertebral body soft tissue, the morphology and signal changes of the vertebral body veins (*P* < 0.05). The apparent diffusion coefficient (ADC) value of the benign group was higher than that of the malignant group (*P* < 0.01). The combined diagnosis of CT and MRI demonstrated superior accuracy, sensitivity, and specificity than the individual diagnoses of CT and MRI (*P* < 0.05).

**Conclusion:**

The combined use of MRI and CT exhibits relatively high accuracy, sensitivity, and specificity in differentiating the nature of vertebral compression fractures, and it has a good value in the identification of fracture types.

## Introduction

Vertebral compression fractures (VCFs) are relatively common in clinical practice (1). With the intensification of the global aging problem, the incidence of osteoporosis has significantly increased, leading to a corresponding rise in the incidence of VCFs (2, 3). According to the cause, VCFs can be classified into two major categories: benign fractures and malignant fractures (4). Benign fractures are mainly caused by osteoporosis and trauma, while malignant fractures are usually caused by primary or metastatic tumors (5). There are significant differences in treatment methods and prognosis between these two types of fractures. For instance, benign fractures are typically treated with conservative therapy or minimally invasive surgery, while malignant fractures may require more aggressive treatment methods such as radiotherapy, chemotherapy, or complex surgeries (6, 7). Therefore, accurately differentiating between benign and malignant VCFs is essential in clinical practice.

In clinical diagnosis, magnetic resonance imaging (MRI) and computed tomography (CT) are two commonly applied imaging examination methods (8, 9). MRI generates images through detecting the behavior of hydrogen atomic nuclei in the magnetic field within tissues, providing high-resolution contrast for soft tissues and having unique advantages in detecting bone marrow edema, soft tissue injuries, and tumors (10). CT uses X-rays to generate images based on the attenuation differences of different tissues, offering clearer display of bone structures, and is particularly suitable for detecting fracture lines and cortical bone destruction (11). These two methods have a crucial role in clarifying the causes of VCFs and assisting clinicians in formulating treatment plans.

Although studies have shown that MRI and CT have relatively clear roles in diagnosing benign and malignant VCFs, there are relatively few clinical studies on the combined application of these two methods for differential diagnosis. Currently, when clinical doctors encounter complex cases, they often rely on personal experience and comprehensive judgment, which may lead to inconsistent and uncertain diagnoses. Moreover, a single imaging examination method may have limitations, such as MRI's less clear display of calcification compared to CT, and CT's inferior ability to distinguish soft tissues compared to MRI (12). Therefore, exploring the diagnostic efficacy of the combined application of MRI and CT is of great significance for further improving the diagnostic accuracy of benign and malignant VCFs.

In view of this, this study aimed to explore the clinical value of utilizing MRI in conjunction with CT for the differential diagnosis of VCFs, with the expectation of providing clinicians with more accurate and reliable diagnostic evidence, thereby optimizing treatment plans and improving patient prognosis.

## Material and methods

### Patients

From January 2020 to January 2025, 150 patients with single VCFs who were admitted to our hospital were selected as study participants. According to the pathological findings, they were divided into the benign group of benign VCFs (76 cases) and the malignant group of malignant VCFs (74 cases). This study was approved by the hospital's ethics committee. All patients and their families signed the informed consent form, voluntarily participating in this research.

Inclusion criteria: (1) clinically diagnosed as VCFs; (2) all the enrolled patients underwent both CT and MRI examinations; (3) all patients, irrespective of the initial clinical or imaging suspicion, underwent percutaneous needle biopsy of the affected vertebra to establish a definitive pathological diagnosis, which served as the reference standard. Exclusion criteria: (1) patients with a severe history of vertebral trauma; (2) those with severely impaired heart, liver, and kidney functions; (3) those with claustrophobia; (4) patients with severe mental disorders.

### Examination methods

MRI examination: all patients were examined using the PHILIPS Ingenia CX 3.0T MRI from Philips (Netherlands), with a spinal coil. The patient was in a supine position, with the head facing forward, and the vertebrae were centered for scanning. Routine T_1_-weighted imaging (T_1_WI), T_2_-weighted imaging (T_2_WI), T_2_W fat-suppressed sagittal imaging, and diffusion weighted imaging (DWI) were performed. Sagittal scanning was carried out using the TSE sequence. T_2_WI-fs parameters: repetition time (TR) 3,050 ms, echo time (TE) 58 ms, FOV 320 mm × 320 mm, slice thickness 4 mm, slice spacing 0.8 mm, excitation 1 time, 15 layers collected, *b* value set to 0 and 800 s/mm^2^, and the apparent diffusion coefficient (ADC) map was obtained. Two board-certified radiologists, each with over 10 years of experience in musculoskeletal imaging, independently reviewed all MRI and CT images. Both radiologists were blinded to the final pathological diagnosis (the reference standard) and to each other's interpretations throughout the entire image evaluation process. Any discrepancies in their initial assessments were resolved through a subsequent consensus discussion, which was also conducted without knowledge of the pathological results. Two radiologists reviewed the images to determine the affected vertebra segments, select the region of interest (ROI), and measure the ADC value of the affected vertebra.

CT examination: all patients underwent spiral CT scans using the GE BrightSpeed 16-slice CT scanner (General Electric, USA). No intravenous contrast agent was administered. The scanning parameters were as follows: slice thickness 5 mm, pitch 1.0–1.5, field of view 18 cm, and the scanning range extended from the affected vertebra to the adjacent vertebrae above and below.

### Observation indicators

MRI signs: (1) degree of vertebral body compression: degree I (< 1/3), degree II (1/3–2/3), degree III (>2/3); (2) extent of vertebral body lesion (on MRI): G1 (< 1/4 and no abnormal signal area in the vertebral body was present), G2 (1/4–2/4 and the distribution pattern of the vertebral body lesion was localized and scattered), G3 (2/4–3/4 and uniform infiltration of low signal), G4 (entirely invaded); this grading (G1–G4) describes the proportion of the vertebral body's bone marrow space that demonstrates abnormal signal intensity on MRI, which may correspond to pathologic processes such as edema, hemorrhage, or tumor infiltration; (3) location of the lesion (adjacent to the endplates or anterior, posterior margins of the vertebral body); (4) post-compression state of the vertebrae (normal or depressed, outward bulging); (5) soft tissues surrounding the vertebrae; (6) vertebral pedicle morphology (invasion, no invasion, not clearly visible); (7) changes after enhancement (flocculent enhancement, nodular strengthening); (8) alterations in the signal of the compressed vertebrae (high signal in DWI, low signal in DWI, low signal in T_1_WI, low signal in T_2_WI).

ADC value: the ADC value was measured using the ROI method. A standardized rectangular ROI with an area of 35 mm^2^ (approximately 5 mm × 7 mm) was consistently placed within the central region of the fractured vertebral body on the sagittal ADC map. The placement adhered to the following criteria: (1) positioned parallel to the vertebral endplates; (2) avoiding cortical margins, visible blood vessels, and artifacts; (3) encompassing the most representative area of abnormal signal within the fracture zone. For each patient, the ADC value was measured three times, and the average was recorded. The same ROI placement protocol was applied to measure the ADC value of an adjacent normal vertebra for comparison. A schematic representation of the standardized ROI placement on the sagittal ADC map for measurement in both fractured and normal vertebrae is provided in Supplementary Figure S1.

The diagnose accuracy rate, specificity and sensitivity of CT, MRI and combined detection in differentiating VCFs were compared. The diagnostic consistency was evaluated using the Kappa test. A Kappa value of 0.8–1.00 represents excellent consistency, a value of 0.6–0.8 represents high consistency, a value of 0.4–0.6 represents moderate consistency; a value of 0.2–0.4 represents general consistency; and a value of 0.0–0.2 represents poor consistency.

### Statistical methods

SPSS version 23.0 (IBM Corp., Armonk, NY, USA) statistical software to implement statistical analysis and processing of the obtained data. Measurement data that met the normal distribution were expressed as mean ± standard deviation (x ± s), and the comparison was conducted using the *t*-test. Count data were expressed as percentages (%), and the comparison was conducted using the χ^2^ test. A difference was considered statistically significant when *P* < 0.05.

## Results

### Baseline characteristics

No significant differences were seen in gender, age and BMI between the two groups (*P* > 0.05, Table 1).

**Table 1:** Comparison of baseline data between the two groups.

**Baseline characteristics**	**Benign group (*n* = 76)**	**Malignant group (*n* = 74)**	***χ^2^/t***	***P-*value**
**Gender**			0.257	0.611
Male	40 (52.63)	42 (56.76)		
Female	36 (47.37)	32 (43.24)		
Age (years)	52.75 ± 5.57	53.26 ± 5.65	0.556	0.578
BMI (kg/m^2^)	23.91 ± 2.95	23.64 ± 2.83	0.571	0.568

### Compression degree and extent of vertebral body lesion

**Figure 1:**
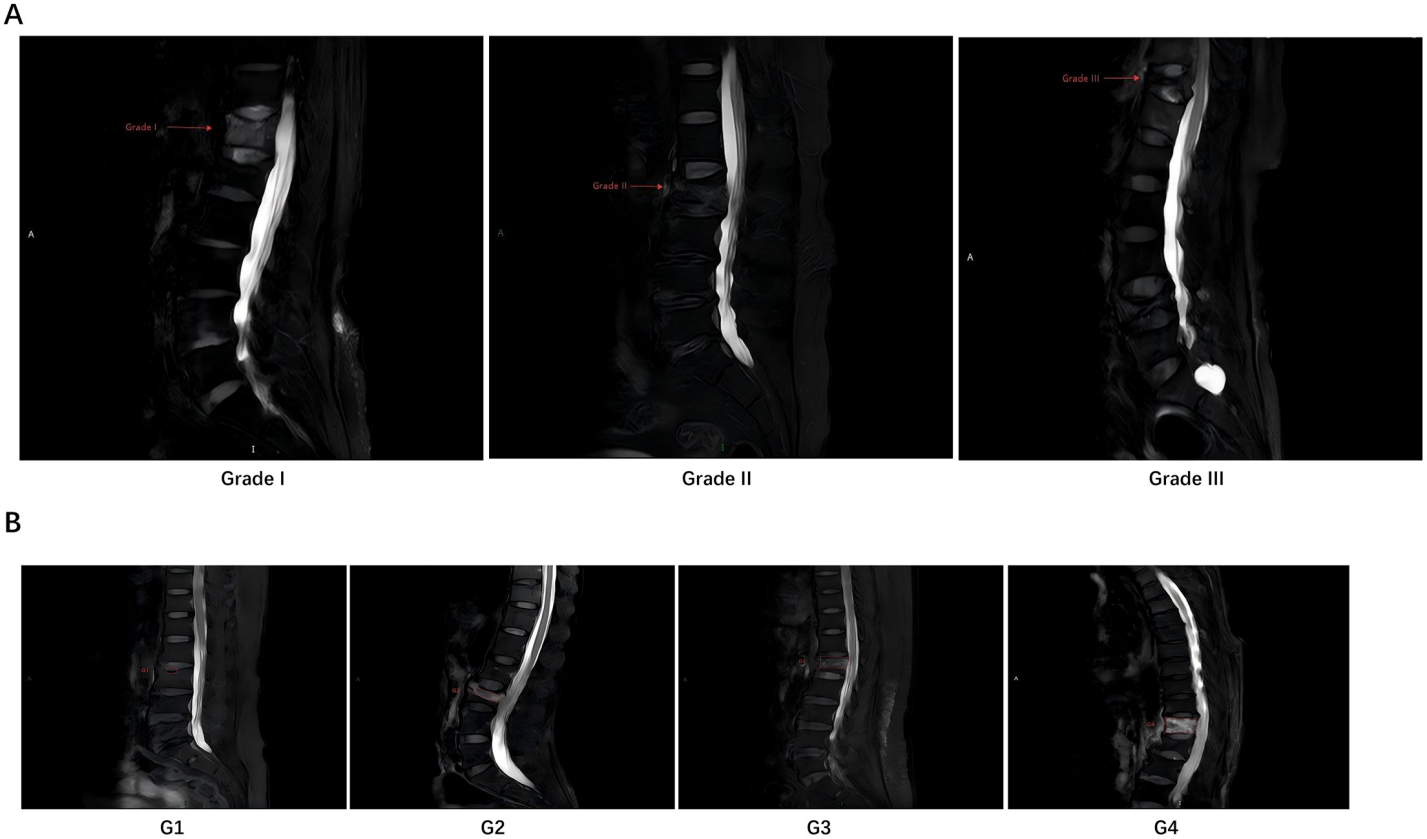
Illustrative examples of MRI grading for vertebral compression fractures. **(A)** Demonstrates the grading of vertebral body compression (Grade I, II, III) on sagittal T2-weighted images with arrows indicating the compression fraction. **(B)** Demonstrates the grading of vertebral body lesion extent (G1, G2, G3, G4) on sagittal T1-weighted or STIR images, with dotted lines outlining the approximate involved area.

As shown in Table 2, in the benign group, there were 10 cases with degree I bone compression at the fracture site, 41 cases with degree II, and 25 cases with degree III; in the malignant group, there were 57 cases with degree I bone compression at the fracture site, seven cases with degree II, and 10 cases with degree III. There was a significant difference in the degree of bone compression between the two groups (*P* < 0.001). In the benign group, there were 26 cases with lesion range of G1, 25 cases with G2, 12 cases with G3, and 13 cases with G4; in the malignant group, there were 12 cases with lesion range of G1, eight cases with G2, 17 cases with G3, and 37 cases with G4. The malignant lesions were more frequently found throughout the entire vertebrae, and the difference between the two groups was significant (*P* < 0.001). Representative MR images illustrating the grading systems for vertebral body compression degree and lesion extent are provided in Figure 1.

**Table 2:** Comparison of compression degree and extent of vertebral body lesion between the two groups.

**MRI signs**	**Benign group (*n* = 76)**	**Malignant group (*n* = 74)**	***χ^2^***	***P-*value**
**Compression degree**			63.466	< 0.001
Degree I	10 (13.16)	57 (77.03)		
Degree II	41 (53.95)	7 (9.46)		
Degree III	25 (32.89)	10 (13.51)		
**Lesion range**			26.275	< 0.001
G1	26 (34.21)	12 (16.22)		
G2	25 (32.89)	8 (10.81)		
G3	12 (15.79)	17 (22.97)		
G4	13 (17.11)	37 (50.00)		

### Location of lesions, the post-compression state of the vertebrae, the soft tissues surrounding the vertebrae, and the vertebral vein morphology

The lesion locations of patients in both groups were mostly adjacent to the endplates. The vertebral bodies were mostly normal or depressed. The number of cases with outward bulging in the benign group was less than that in the malignant group, and the number of cases with soft tissue invasion around the vertebral bodies was also less in the benign group than in the malignant group. The morphology of the vertebral veins was mostly not invaded. Comparisons of the lesion locations, the post-compression states of the vertebral bodies, the soft tissue surrounding the vertebral bodies, and the morphology of the vertebral veins between the two groups showed statistically significant differences (*P* < 0.05, Table 3).

**Table 3:** Comparison of the location of lesions, the post-compression state of the vertebrae, the soft tissues surrounding the vertebrae, and the vertebral vein morphology between the two groups.

**MRI signs**	**Benign group (*n* = 76)**	**Malignant group (*n* = 74)**	***χ^2^***	***P*-value**
**Location of lesions**			8.176	0.004
Adjacent to the endplates	74 (97.37)	62 (83.78)		
Anterior and posterior margins of the vertebral body	2 (2.63)	12 (16.22)		
**Post-compression state of the vertebrae**			4.740	0.029
Normal or depressed	63 (82.89)	50 (67.57)		
Outward bulging	13 (17.11)	24 (32.43)		
**Soft tissues surrounding the vertebrae**			30.093	< 0.001
Yes	7 (9.21)	37 (50.00)		
No	69 (90.79)	37 (50.00)		
**Vertebral vein morphology**			10.382	0.005
Not invaded	60 (78.95)	42 (56.76)		
Invaded	4 (5.26)	15 (20.27)		
Not clearly visible	12 (15.79)	17 (22.97)		

### Morphology of pedicles, changes after enhancement, and alterations in the signal of the compressed vertebrae

The number of cases with normal signal intensity of the pedicle in the benign group was greater than that in the malignant group, and the number of cases with destruction was less than that in the malignant group. The number of cases with flocculent enhancement of the lesion in the benign group was higher than that in the malignant group, while the number of cases with nodular enhancement was lower than that in the malignant group. In terms of the number of cases showing signal changes after enhanced scanning (except for the low signal in DWI, which was weaker than that in the malignant group), the benign group had a higher number than the malignant group. Comparisons of the morphology of the pedicle, the changes after enhancement, and the signal changes of the compressed vertebrae between the two groups showed significant differences (*P* < 0.001, Table 4).

**Table 4:** Comparison of the morphology of pedicles, changes after enhancement, and alterations in the signal of the compressed vertebrae between the two groups.

**MRI signs**	**Benign group (*n* = 76)**	**Malignant group (*n* = 74)**	***χ^2^***	***P-*value**
**Morphology of pedicles**			83.705	< 0.001
Low signal on T_1_WI	59 (77.63)	3 (4.05)		
Destruction	17 (22.37)	71 (95.95)		
**Changes after enhancement**			116.180	< 0.001
Flocculent enhancement	71 (93.42)	4 (5.41)		
Nodular enhancement	5 (6.58)	70 (94.59)		
**Alterations in the signal of the compressed vertebrae**			87.130	< 0.001
High signal in DWI	70 (92.11)	12 (16.22)		
Low signal in DWI	6 (7.89)	62 (83.78)		
Low signal in T_1_WI	71 (93.42)	47 (63.51)	19.983	< 0.001
Low signal in T_2_WI	73 (96.05)	34 (45.94)	46.033	< 0.001

### ADC value

**Figure 2:**
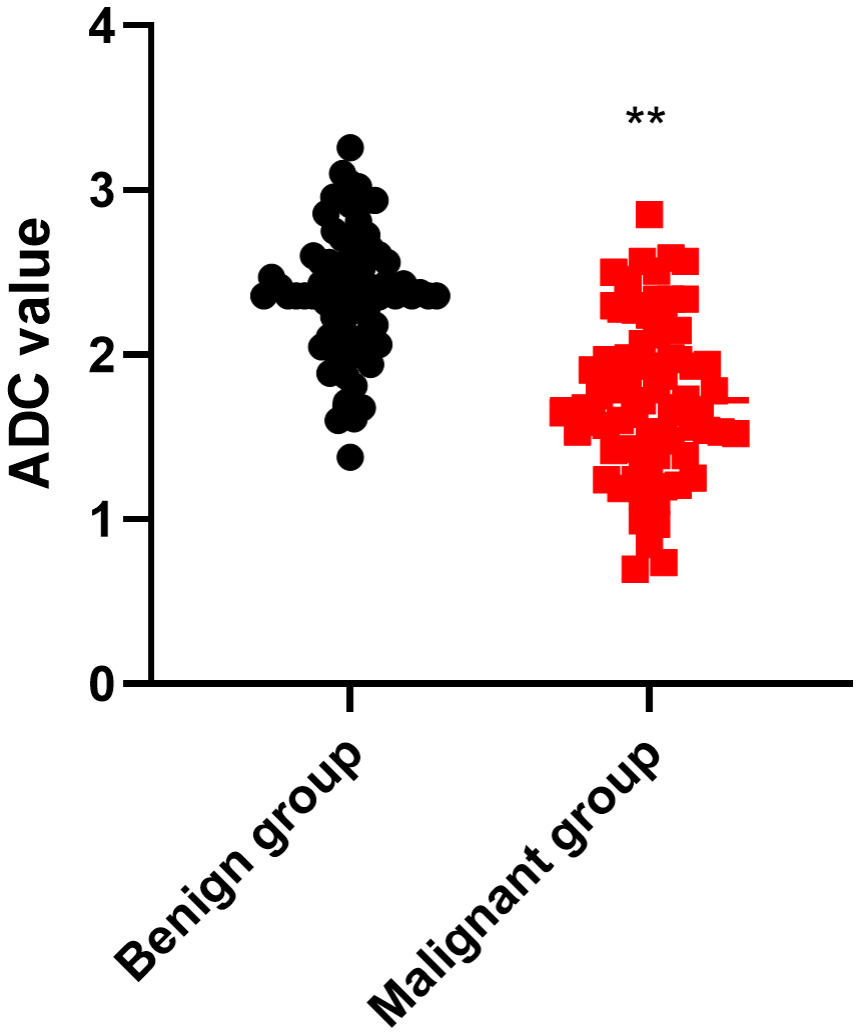
Comparison of ADC value between the two groups. **P < 0.01.

As shown in Figure 2, the ADC value of the benign group was higher than that of the malignant group (*P* < 0.01).

### Efficacy of CT, MRI and combined detection in differentiating VCFs

The cross-tabulation of imaging diagnoses against the pathological standard for each modality is presented in Table 5. As shown in Tables 5, 6, CT demonstrated a diagnostic accuracy of 90.00% for benign VCFs, with a sensitivity of 89.47%, specificity of 90.54%, positive predictive value of 90.67%, negative predictive value of 89.33%, and a Kappa value of 0.87. In comparison, MRI showed an accuracy of 92.67%, sensitivity of 92.11%, specificity of 93.24%, positive predictive value of 93.33%, negative predictive value of 92.00%, and a Kappa value of 0.85. When CT and MRI were combined for diagnosis, the accuracy improved significantly to 96.67%, with sensitivity reaching 96.05%, specificity at 97.30%, positive predictive value at 97.33%, negative predictive value at 96.00%, and a Kappa value of 0.92. Notably, the combined CT + MRI approach exhibited superior diagnostic accuracy compared to using either CT or MRI alone (*P* < 0.05).

**Table 5:** Diagnostic performance of CT, MRI, and combined CT+MRI in differentiating benign and malignant VCFs compared to pathological diagnosis.

**Examination method**	**Diagnosis by imaging**	Pathological diagnosis (gold standard)		**Total diagnoses by modality**
		**Benign (*****n*** = **76)**	**Malignant (*****n*** = **74)**	
CT	Benign	68	7	75
	Malignant	8	67	75
MRI	Benign	70	5	75
	Malignant	6	69	75
Combined CT+MRI	Benign	73	2	75
	Malignant	3	72	75

The table displays the cross-tabulation of imaging diagnoses against the pathological gold standard for each imaging modality (CT, MRI, and their combination). The “Total Diagnoses by Modality” column sums the number of cases diagnosed as benign or malignant by that specific imaging approach. Since each of the 150 patients received a single diagnosis (benign or malignant) from each modality, the row totals for “Benign” and “Malignant” diagnoses under each modality sum to 150 (e.g., for CT: 75 benign diagnoses + 75 malignant diagnoses = 150 total diagnoses, matching the patient number).

**Table 6:** Diagnostic efficacy of CT, MRI, and combined CT+MRI in differentiating benign and malignant VCFs.

**Examination method**	**Accuracy rate**	**Sensitivity**	**Specificity**	**Positive predictive value (PPV)**	**Negative predictive value (NPV)**	**Kappa value**
CT	90.00% (135/150)	89.47% (68/76)	90.54% (67/74)	90.67% (68/75)	89.33% (67/75)	0.87
MRI	92.67% (139/150)	92.11% (70/76)	93.24% (69/74)	93.33% (70/75)	92.00% (69/75)	0.85
Combined CT+MRI	96.67% (145/150)	96.05% (73/76)	97.30% (72/74)	97.33% (73/75)	96.00% (72/75)	0.92

Sensitivity = True Positives/(True Positives + False Negatives); denominator is the total number of pathologically confirmed benign cases (*n* = 76). Specificity = True Negatives/(True Negatives + False Positives); denominator is the total number of pathologically confirmed malignant cases (*n* = 74). Accuracy = (True Positives + True Negatives)/Total Cases (*n* = 150). PPV = True Positives/(True Positives + False Positives); NPV = True Negatives/(True Negatives + False Negatives). The denominators for PPV and NPV are the total number of cases diagnosed as benign or malignant by the imaging modality, respectively, as derived from Table 5.

## Discussion

VCFs are classified as benign fractures and malignant fractures based on the cause (13). Clinically, by analyzing CT and MRI, the imaging characteristics of benign and malignant VCFs have been examined, and it has been confirmed that these features have a high specificity for distinguishing between benign and malignant VCFs. MRI has high resolution and sensitivity, and can utilize multi-parameter imaging to observe the subtle pathological changes in soft tissues and bone marrow tissues (14). CT can distinguish the degree of bone destruction and the fine structure of vertebrae by observing the vertebral body, the density of bone trabeculae, and the anterior and posterior margins of the vertebral body (15).

Osteoporotic VCFs are benign fractures. The CT images indicate that the reduction of bone components is the main cause of osteoporotic VCFs. At the same time, the bone marrow shows no significant change. When the fracture occurs, the bone marrow shifts along the direction of compression, maintaining the smoothness and normal shape of the vertebral edge and morphology. The CT imaging features of malignant fractures are multiple jumping vertebral involvement and significant involvement of the vertebral appendages (16). The MRI manifestations of osteoporotic VCFs include a reduction in the paravertebral soft tissue, posterior displacement of the vertebral body's lower or upper part, and changes in the T_1_WI low signal and T_2_WI high signal bone marrow edema signals under or within the vertebral endplate or the central part of the vertebral body. These changes are uniform and consistent with the normal changes of the vertebral body (17).

Malignant VCFs occur when the tumor completely erodes the vertebral body, resulting in weakened bone trabeculae or cortical structure, as well as various pathological changes such as invasive growth, degeneration, necrosis, hemorrhage, and edema around the fracture line (18). The MRI of malignant fractures shows that the T_1_WI and T_2_WI signals in the central part of the vertebral body or the adjacent area are disordered and unevenly changed, and are higher than the normal vertebral body signals. The boundaries of the bone marrow affected by malignant lesions are irregular, and the paravertebral soft tissues enlarge. Therefore, the T_1_WI bone marrow signals show significant differences, which can be used as a criterion for differentiating benign and malignant conditions (19). While extensive vertebral body involvement (G4 range) was a strong indicator of malignancy in our cohort, it is noteworthy that 13 cases (17.1%) of pathologically confirmed benign fractures also exhibited this pattern. This underscores the potential for imaging overlap and the risk of false-positive diagnosis. In these benign G4 cases, the diffuse signal abnormality likely resulted from severe bone marrow edema and hemorrhage secondary to acute, high-energy trauma or in the context of profound osteoporosis with extensive micro-fractures and reactive changes. To avoid misclassifying such benign lesions as malignant, clinicians and radiologists should prioritize a constellation of ancillary imaging features rather than relying on a single sign. Key discriminators favoring a benign etiology in the setting of G4 involvement include: (1) preservation of the vertebral pedicles without osteolytic destruction (as opposed to common pedicle involvement in metastases); (2) absence of an associated paravertebral soft-tissue mass; (3) a flocculent or diffuse, non-nodular enhancement pattern post-contrast, typical of reactive hyperemia rather than tumor neovascularity; (4) higher ADC values on DWI, reflecting free water diffusion in edematous marrow rather than restricted diffusion in cellular tumors. The integration of these multiparametric features is crucial for accurate differentiation. In patients with malignant VCFs, due to the infiltration of tumor cells into the bone marrow, the extracellular space becomes narrower, restricting the movement of water molecules and slowing down the diffusion rate, resulting in a decrease in ADC values (20). However, in patients with benign VCFs, the bone marrow is congested and edematous, allowing water molecules to move freely in the extracellular space, accelerating the diffusion process and causing an increase in ADC values (21).

The results of this study demonstrated, among patients with osteoporotic VCFs, the number of cases with degree I compression was less than that of patients with malignant VCFs, while the number of cases with degrees II and III was higher than that of patients with malignant VCFs. The extent of lesion involvement in patients with benign VCFs showed lower than that in patients with malignant VCFs. The lesion locations of patients were mostly close to the endplates, and the vertebrae were mostly normal or depressed. Patients with benign VCFs had fewer cases of outward bulging compared to those with malignant VCFs, and the cases of surrounding soft tissue involvement were also fewer than those with malignant VCFs. The morphology of the patient's vertebral veins was mostly not affected. In benign VCFs, the number of cases with normal pedicle signals and the number of cases with fluffy-like enhancement of the lesion after enhancement were higher in patients with benign VCFs than in patients with malignant VCFs. The number of cases with destruction, the number of nodular enhancement cases, and the signal changes in the posterior part of the compressed vertebrae, except for the low signal on DWI, were less in patients with benign VCFs than in patients with malignant VCFs. Meanwhile, the ADC value of patients with benign VCFs was higher than that of patients with malignant VCFs.

An interesting observation in our study was that the inter-observer agreement, as measured by the Kappa statistic, was slightly higher for CT alone (κ = 0.87) than for MRI alone (κ = 0.82), despite MRI's superior soft-tissue contrast. This may be attributable to several factors. First, the evaluation of malignant involvement on CT primarily relies on more objective and dichotomous signs, such as the presence or absence of cortical destruction, fracture lines extending to the posterior cortex, and pedicle osteolysis. These features often have clearer demarcation. In contrast, MRI assessment involves interpreting more continuous and potentially subtle signal intensity changes (e.g., gradations of bone marrow edema, heterogeneous enhancement patterns) and morphological alterations in soft tissues, which can introduce greater subjectivity and interpretive variability between readers. Second, severe bone marrow edema in acute benign fractures can sometimes mimic the diffuse infiltration pattern seen in malignancies on MRI, leading to diagnostic uncertainty and lower agreement. The combination of CT and MRI effectively mitigates these limitations by providing complementary structural and contrast-based information, thereby achieving the highest level of inter-observer concordance (κ = 0.92).

Consistently, it has been reported that the ADC may be an effective in distinguishing benign and malignant VCFs (22). Additionally, our study indicated that the diagnostic coincidence rate, specificity, and sensitivity of CT combined with MRI were all higher than those of the two examinations alone. Consistently, Aggarwal et al. suggested that the combined use of MRI and positron emission tomography-CT demonstrated a 100% specificity in distinguishing between benign and malignant lesions associated with VCFs (23).

Translating our findings into clinical practice requires consideration of diagnostic efficiency and resource allocation. While our data robustly support the superior diagnostic accuracy of the combined CT+MRI approach, a pragmatic, risk-stratified imaging strategy may be considered. For patients with a high pre-test probability of benign etiology (e.g., clear history of trauma, known severe osteoporosis without systemic symptoms), an initial MRI may provide sufficient diagnostic confidence, given its high sensitivity for marrow edema and fracture detection. Conversely, for patients with red flags suggestive of malignancy (e.g., unexplained weight loss, nocturnal pain, history of primary cancer, or atypical MRI features), proceeding directly to a combined CT+MRI protocol could be justified to maximize diagnostic certainty and expedite treatment planning. In resource-limited settings or when MRI is contraindicated, CT serves as a valuable initial tool; if its findings are equivocal or suspicious, MRI should be subsequently employed. Future prospective studies are warranted to validate such workflow models and to formally evaluate their cost-effectiveness and impact on patient outcomes.

This study has several limitations that should be considered when interpreting the results. First, it was a retrospective, single-center study conducted at a specialized hospital. The patient population and imaging protocols may not be fully representative of other clinical settings or diverse demographic groups, which could affect the generalizability of our findings. Second, while the diagnostic accuracy of the combined imaging approach was our primary endpoint, the study did not assess long-term patient outcomes, such as survival, treatment response, or quality of life, following the application of this diagnostic strategy. Third, from a technical standpoint, although our MRI protocol was comprehensive, the CT scans were performed without intravenous contrast. The use of contrast-enhanced CT might have provided additional value in characterizing paravertebral soft tissue involvement. Furthermore, advanced CT reconstructions or dual-energy CT techniques were not employed, which might offer improved bone marrow assessment. Fourth, our study design focused on diagnostic performance and was not intended to evaluate the cost-effectiveness, optimal sequencing, or real-world workflow integration of performing both CT and MRI. The pragmatic imaging pathway suggested in the discussion remains speculative and requires formal validation in prospective health-economic and implementation studies. Finally, the sample size, though adequate for the primary analysis, may limit the power for subgroup analyses of specific fracture etiologies or rare malignant subtypes.

## Conclusion

The combination of CT and MRI has a high diagnostic agreement rate, sensitivity and specificity in evaluating the benign and malignant nature of VCFs. It has significant diagnostic significance in the clinical diagnosis of VCFs.

## Data Availability

The datasets presented in this study can be found in online repositories. The names of the repository/repositories and accession number(s) can be found in the article/Supplementary material.

## References

[1] Kutsal FY Ergin Ergani GO. Vertebral compression fractures: still an unpredictable aspect of osteoporosis. Turk J Med Sci. (2021) 51:393–9. doi: 10.3906/sag-2005-315 32967415 PMC8203169

[2] Wang L Yu W Yin X Cui L Tang S Jiang N . Prevalence of osteoporosis and fracture in China: the China Osteoporosis Prevalence Study. JAMA Netw Open. (2021) 4:e2121106. doi: 10.1001/jamanetworkopen.2021.21106 34398202 PMC8369359

[3] Xiao PL Cui AY Hsu CJ Peng R Jiang N Xu XH . Global, regional prevalence, and risk factors of osteoporosis according to the World Health Organization diagnostic criteria: a systematic review and meta-analysis. Osteoporos Int. (2022) 33:2137–53. doi: 10.1007/s00198-022-06454-3 35687123

[4] Liu B Jin Y Feng S Yu H Zhang Y Li Y. Benign vs malignant vertebral compression fractures with MRI: a comparison between automatic deep learning network and radiologist's assessment. Eur Radiol. (2023) 33:5060–8. doi: 10.1007/s00330-023-09713-x 37162531

[5] Mauch JT Carr CM Cloft H Diehn FE. Review of the imaging features of benign osteoporotic and malignant vertebral compression fractures. Am J Neuroradiol. (2018) 39:1584–92. doi: 10.3174/ajnr.A5528 29348133 PMC7655272

[6] Ding JK Zhao B Zhai YF. Subsequent fractures after vertebroplasty in osteoporotic vertebral fractures: a meta-analysis. Neurosurg Rev. (2022) 45:2349–59. doi: 10.1007/s10143-022-01755-x 35195800

[7] Sørensen ST Kirkegaard AO Carreon L Rousing R Andersen M. Vertebroplasty or kyphoplasty as palliative treatment for cancer-related vertebral compression fractures: a systematic review. Spine J. (2019) 19:1067–75. doi: 10.1016/j.spinee.2019.02.012 30822527

[8] Alsoof D Anderson G McDonald CL Basques B Kuris E Daniels AH. Diagnosis and management of vertebral compression fracture. Am J Med. (2022) 135:815–21. doi: 10.1016/j.amjmed.2022.02.035 35307360

[9] Kolanu N Silverstone EJ Ho BH Pham H Hansen A Pauley E . Clinical utility of computer-aided diagnosis of vertebral fractures from computed tomography images. J Bone Miner Res. (2020) 35:2307–12. doi: 10.1002/jbmr.4146 32749735

[10] Xing H Liu Z Li Z Liu H Wang Y Chang Z . A radiomics nomogram based on MRI for differentiating vertebral osteomyelitis from vertebral compression fractures. Eur J Radiol. (2025) 187:112106. doi: 10.1016/j.ejrad.2025.112106 40228322

[11] Wiklund P Buchebner D Geijer M. Vertebral compression fractures at abdominal CT: underdiagnosis, undertreatment, and evaluation of an AI algorithm. J Bone Miner Res. (2024) 39:1113–9. doi: 10.1093/jbmr/zjae096 38900913

[12] Li YB Zheng X Wang R Wu H Han S Deng ZY . SPECT-CT versus MRI in localizing active lesions in patients with osteoporotic vertebral compression fractures. Nucl Med Commun. (2018) 39:610–7. doi: 10.1097/MNM.0000000000000857 29893749

[13] Bacher S Hajdu SD Maeder Y Dunet V Hilbert T Omoumi P. Differentiation between benign and malignant vertebral compression fractures using qualitative and quantitative analysis of a single fast spin echo T2-weighted Dixon sequence. Eur Radiol. (2021) 31:9418–27. doi: 10.1007/s00330-021-07947-1 34041569 PMC8589814

[14] Cicala D Briganti F Casale L Rossi C Cagini L Cesarano E . Atraumatic vertebral compression fractures: differential diagnosis between benign osteoporotic and malignant fractures by MRI. Musculoskelet Surg. (2013) 97(Suppl 2):S169–79. doi: 10.1007/s12306-013-0277-9 23949939

[15] Petritsch B Kosmala A Weng AM Krauss B Heidemeier A Wagner R . Vertebral compression fractures: third-generation dual-energy CT for detection of bone marrow edema at visual and quantitative analyses. Radiology. (2017) 284:161–8. doi: 10.1148/radiol.2017162165 28240561

[16] Li C Lai XM Liu N Lin Y Hu W. Correlation analysis of the vertebral compression degree and CT HU value in elderly patients with osteoporotic thoracolumbar fractures. J Orthop Surg Res. (2023) 18:457. doi: 10.1186/s13018-023-03941-z 37365576 PMC10294538

[17] Qi H Qi J Sun Y Gao J Sun J Wang G. Value of MRI in assessing back pain after thoracolumbar osteoporotic vertebral compression fractures and discussion on the underlying mechanisms by tissue biopsy. Eur Spine J. (2022) 31:1147–57. doi: 10.1007/s00586-021-07095-6 35038034

[18] McDonald CL Alsoof D Daniels AH. Vertebral compression fractures. R I Med J. (2022) 105:40–5. 36173908

[19] Tanlak E Özpar R Tobcu E Inecikli MF Nas ÖF Hakyemez B. Multiparametric spinal MRI for differentiating benign and malignant vertebral compression fractures. Radiologie. (2025) 65:106–15. doi: 10.1007/s00117-025-01512-3 40982034

[20] Maeda M Sakuma H Maier SE Takeda K. Quantitative assessment of diffusion abnormalities in benign and malignant vertebral compression fractures by line scan diffusion-weighted imaging. Am J Roentgenol. (2003) 181:1203–9. doi: 10.2214/ajr.181.5.1811203 14573404

[21] Rumpel H Chong Y Porter DA Chan LL. Benign versus metastatic vertebral compression fractures: combined diffusion-weighted MRI and MR spectroscopy aids differentiation. Eur Radiol. (2013) 23:541–50. doi: 10.1007/s00330-012-2620-1 22903620

[22] Wonglaksanapimon S Chawalparit O Khumpunnip S Tritrakarn SO Chiewvit P Charnchaowanish P. Vertebral body compression fracture: discriminating benign from malignant causes by diffusion-weighted MR imaging and apparent diffusion coefficient value. J Med Assoc Thai. (2012) 95:81–7. 22379746

[23] Aggarwal A Salunke P Shekhar BR Chhabra R Singh P Bhattacharya A . The role of magnetic resonance imaging and positron emission tomography-computed tomography combined in differentiating benign from malignant lesions contributing to vertebral compression fractures. Surg Neurol Int. (2013) 4:S323–6. doi: 10.4103/2152-7806.112619 23878766 PMC3717528

