# Supplementary figures and images for: Diagnostic value of computed tomography plus magnetic resonance imaging in assessing the benign and malignant nature of vertebral compression fractures
Source: Front Med (Lausanne). 2026 Mar 9;13:1759764. doi: 10.3389/fmed.2026.1759764 (PMC13006301; doi:10.3389/fmed.2026.1759764)

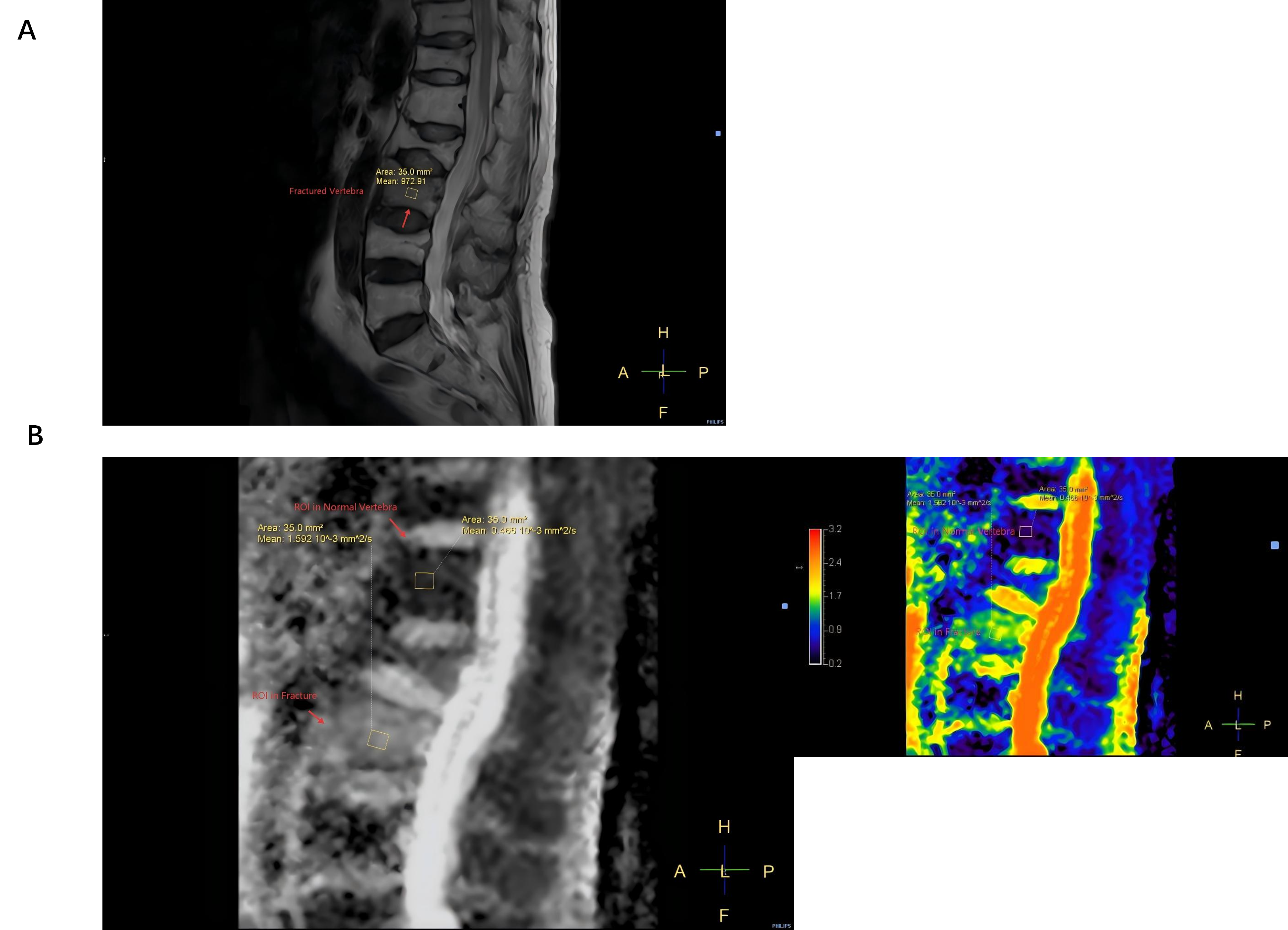

Supplement: Supplementary file 1 [file Image_1.tif]
